# Are Citric Acid-Iron II Complexes True Chelates or Just Physical Mixtures and How to Prove This?

**DOI:** 10.3390/foods12020410

**Published:** 2023-01-15

**Authors:** Ghadeer Mattar, Amira Haddarah, Joseph Haddad, Montserrat Pujola, Francesc Sepulcre

**Affiliations:** 1 Departament d’Enginyeria Agroalimentària i Biotecnologia, Universitat Politècnica de Catalunya, Campus del Baix Llobregat, Carrer Esteve Terradas 8, Castelldefels, 08860 Barcelona, Spain; 2 Doctoral School of Sciences and Technology, Lebanese University, Rafic Hariri Campus, Hadath, Baabda 1533, Lebanon

**Keywords:** chelation, citric acid, iron chelates, NIR, HPLC, molar ratio

## Abstract

Although mineral chelates are widely produced to be used as food fortifiers, the proof that these complexes are chelates is still missing. In our present work, iron II complexes using citric acid in different ratios are produced, and the occurrence of chelation is investigated along with its behavior according to a molar ratio between the ligand and the mineral. High performance liquid chromatography (HPLC), flame atomic absorption spectroscopy (FAAS), ultraviolet-visible spectroscopy (UV-Vis), Fourier-transform infrared (FTIR), and near infrared spectroscopy (NIR) were used for a non-structural characterization of these complexes. In contrast to published work, our findings show that the chelation of citric acid is achieved in the liquid form and at a low pH and that the molar ratio is very important in setting the direction of the reaction, either toward chelation or dimer formation. The ratio citric acid:iron 1:4 seems to be the most convenient ratio in which no free citric acid remains in the solution, while the 1:3 ratio behaves differently, requiring further investigations by such techniques as extended X-ray absorption fine structure spectroscopy (EXAFS), among others, in order to deeply identify the structural organization occurring in this ratio. NIR, extensively used in industries, proved to be very useful in the demonstration and characterization of chelates. These findings are particularly advantageous for pharmaceutical and food industries in offering an innovative competent fortifying agent to be used in combatting iron deficiency.

## 1. Introduction

Food insecurity is increasing worldwide despite the continuous efforts of the governments and organizations to combat it. A non-COVID-19 scenario was assumed in the projection of undernourishment in 2030, in which the number of undernourished people is expected to be 841.4 million, whereas when taking the COVID-19 pandemic into consideration, the optimistic scenario will be 860.3 million of undernourished, and 909 million in the case of the pessimistic scenario [1,2]. Food fortification was found to be a valuable and practical technique for reducing micronutrient deficiencies. Nonetheless, researchers are still extensively investigating the development of new food fortificants with improved properties, which will be able to overcome the limitations of conventional fortifiers. Mineral chelates are considered to be one of the innovative solutions for food fortification problems detailed in a previous review [3]. 

Although mineral chelates using different sequestrants (ethylenediaminetetraacetic acid (EDTA), amino acids, etc.) were widely produced to be used in food fortification, their chemical structure and characteristics are still not clearly presented. The solid proof that these combinations are truly chelates is still missing and most publications suppose the obtained products to be chelates due to the well-known fact of mineral chelation. Indeed, these products might be physical mixtures of reactants or unreacted dry blends [4]. Yunarti et al. [5] produced iron chelates using glycine and used the FTIR spectrum of the obtained chelate to prove chelation by assigning some peaks for Fe-OC (511/cm) and Fe-N (3000–3200/cm) already present in glycine alone. Thus, the evidence relied on to prove chelation does not seem to be solid enough. Whereas Henriksen et al. [6] have used both HPLC and MS techniques to prove the chelation of minerals to amino acids. In HPLC analysis, they intended to extend the time of the analyses so that the third peak corresponding to the complex appeared on higher retention time. Similarly, in mass spectrometry, *m*/*z* peaks corresponding to the molecular weight of the amino acid complexes were identified. However, the authors mentioned that free amino acids were still present with the chelates and were also quantified by HPLC. Although these techniques are highly appreciated for their accuracy and sensitivity, they still have some limitations in chelate analyses. Although HPLC is well-known for its accuracy, the need for harsh chemical treatments and the use of strong solvents in the mobile phase may affect the complex formed or lead to its dissociation. Mass spectrometry, especially ICP-MS, remains to be one of the most reliable methods to prove chelation, characterize and quantify the chelates due to its high sensitivity. However, the equipment, operating, and laboratory setup costs limit its use, especially on the industrial scale. Nonetheless, this technique requires high levels of staff expertise and interferences to be controlled. To be used quantitatively, purification and separation of the chelates must be done prior to analyses; the presence of free metal ions could reduce the accuracy of the analytical method. Moreover, high-temperature ionization destroys the stability of the chelates [7,8]. The use of spectral methods, such as FTIR and NIR, has gained great interest and is being favored, especially in industries. This is due to the fact that most factories nowadays have these instruments and use them for many purposes, specifically in quality control. Moreover, these methods are famous for being efficient, easy to use, non-destructive, able to be incorporated in-line or off-line, and practical in analyzing the sample without any pre-treatment that may affect the results. Finding solid evidence from FTIR and NIR on the occurrence of chelation will greatly help researchers and industries and will enable them to shift toward these feasible techniques.

Citric acid is a weak organic acid present naturally in citrus fruits, and it plays the role of an intermediate in a citric acid cycle. It has the structure of carboxylic acids with three carboxyl groups. It is often used in everyday life and it is a valuable tool for food processors [9,10]. Its use in this field is extensive as a food additive, so it could be also used as a fortifier in combination with minerals to be able to reach at-risk populations. Famous for its sequestering ability, citric acid was chosen to produce citric–iron II chelates in this study instead of iron III citric complexes already present as ferric citrate due to the better assimilation of Fe^2+^ in the human body. Our research aims to prove that chelation has taken place in the produced citric acid–iron compounds, as well as to characterize them quantitatively and qualitatively to be used as innovative food fortificants having optimum characteristics. 

## 2. Materials and Methods

### 2.1. Chemicals and Reagents 

Citric acid (99.5%) and ferrous sulfate heptahydrate (min. 99.0%) were bought from Panreac Quimica SA (Barcelona, Catalonia, Spain).

For sulfate determination, Barium chloride di-hydrate (min. 99%) was bought from POCH (Gliwice, Upper Silesia, Poland), sodium sulfate anhydrous (99.0–100.5%), iso-amyl acetate (99.0%) and NaCl (99%) were bought from Panreac Quimica SA (Barcelona, Catalonia, Spain), 2-propanol (≥99.5%) was bought from JT Baker (Phillipsburg, NJ, USA). 

### 2.2. Preparation of the Chelates

Citric acid–iron chelates were prepared following a protocol similar to that of the production of ferrous bisglycinate followed by Yunarti et al. [5], where anhydrous citric acid and FeSO_4_·7H_2_O were mixed in heat resistant bottles with 100 mL water, flushed with nitrogen gas, and tightly closed. The bottles were then kept at a temperature of about 50 °C with continuous agitation for 24 h. Afterward, they were transferred to the refrigerator for crystallization. Due to the fact that citric acid chelates were not produced before following this manufacturing technique, we have done an optimization step for the molar ratios as well as the solute-to-solvent ratio, four different molar ratios of citric acid/iron solutions were chosen to be prepared and studied. The first ratio of 1:1 was chosen to allow us to study the interaction between citric acid and iron when present in equal amounts, whereas 1:2 and 2:1 were used to study the effect of having double amounts of iron or citric acid, respectively. The last ratio of 2:3 was added to be studied as the ratio in between. The bottles were monitored daily until crystallization occurred. Green crystals were obtained after five days. Iron crystals were then separated from the solutions and left in the open air to dry. The same amount of citric acid and iron sulfate were prepared separately in water following the same procedure. 

After conducting quantitative and qualitative analyses, it was found that other molar ratios within the solubility range of ferrous sulfate were needed to better understand the effect of the molar ratio on chelation and to be able to find the most optimum ratio to be used in citric acid–iron chelation. The same procedure was followed but with a concentration between 0.3–0.75 M and a vast range of ratios of citric acid: iron (1:1, 2:1, 3:1, 4:1, 1:2, 1:3, and 1:4) in the first four samples the amount of iron sulfate was fixed, and the amount of citric acid was increased proportionally, whereas the opposite applied for the last three samples where the citric acid amount was fixed as in 1:1 sample, and the amount of iron sulfate was increased. In this case, we can better compare the samples, and the findings will be due to the reaction occurring without having the effect of concentration leading to precipitation and crystallization. In these seven samples, no crystallization occurred, so the solution was used for analyses.

Part of the solutions of all the samples was dried in the oven at 50 °C (same reaction temperature) until the mass became stable. Further analyses were done for the resulting dried parts. 

### 2.3. Physicochemical Analyses

- The pH value of each solution was recorded before flushing with nitrogen and after removing it from the refrigerator.
- The mass of each iron crystal was recorded before and after drying.
- The volume of each solution was also measured.
- Melting point measurement was done for the commercial products, and for all the solid samples, it was obtained using Stuart™ SMP10 melting point apparatus from Sigma Aldrich. The samples were pulverized and loaded into the closed-end capillary tubes, then placed in the instrument to be heated gradually (2 °C/min); the temperatures were recorded at first drop and also when the sample had turned into liquid [11]. Analysis was done in triplicates.

### 2.4. Spectral and Chromatographic Analysis:

*Atomic absorption spectroscopy (AAS)* was done for the solutions remaining after crystallization and for solutions of iron crystals dissolved in distilled water. These analyses were done to quantify the amount of iron in each sample. Varian SpectrAA-110 Atomic Absorption Spectrophotometer from Agilent Technologies (Chicago, IL, USA) equipped with deuterium lamp background correction, the hallow cathode lamps (HCL), and oxygen-rich air–acetylene flame was used; the conditions to be applied for Fe detection were a wavelength of 248.3 nm, a flow rate of acetylene 1.5 L/min, 3.5 L/min airflow, HCL lamp current of 10 mA, slit width of 0.2 nm, and a measurement time of 8 s [12]. All the samples were diluted to meet the detection limit of the spectroscope, and analysis was done in triplicates.

*UV-Vis spectroscopy* was done for the solutions remaining after crystallization and for a solution of each iron crystal dissolved in distilled water. This technique was used to quantify the amount of sulfates in each sample through the measurement of turbidity following a protocol obtained from [13] and followed by [14] using Thermo Electron Corporation Spectrophotometer type Evolution 300 (Rugby, Warwickshire, UK), at 420 nm in 1cm quartz cuvette. Analysis was done in triplicates.

**Figure 1:**
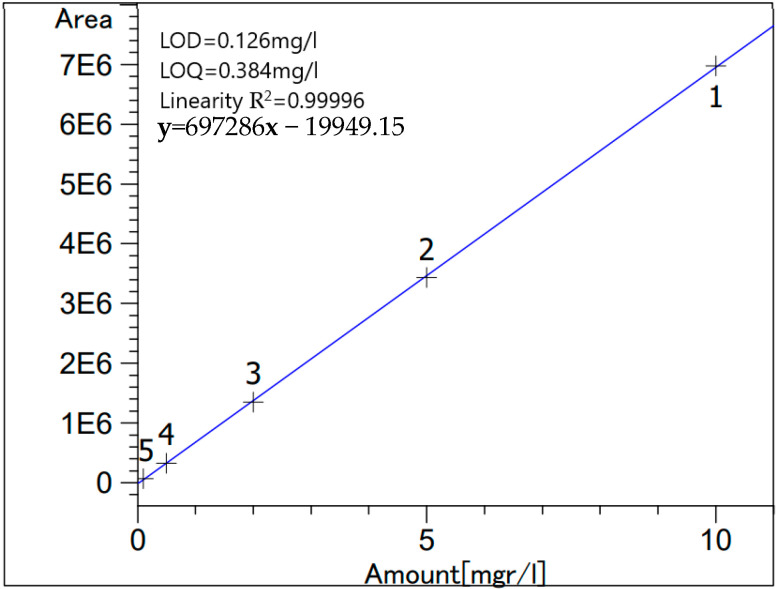
Citric acid standard calibration curve.

*High-performance liquid chromatography (HPLC)* was done for the same samples as AAS in order to quantify the respective amounts of citric acid in each sample. This measurement was done using Beckman (110B, 156 Refractive Index Detector, and C18 columns (Luna 5 µm, 25 cm × 0.4cm), Krefeld, Germany) following the protocol described by Mcfeeters et al. [15]. Stock standard solution of citric acid was prepared by deionized water; then, the various standard solutions were obtained from appropriate dilutions of the stock. Figure 1 and Table 1 show the standard calibration curve and the % recovery, respectively. Analysis was done in triplicates. 

**Infra-Red Spectroscopy:** In order to prove chelation through structural changes, NIR and FTIR were done for samples containing only citric acid or only iron sulfate and for the samples containing both. Due to the high sensitivity of water in the infrared region, the obtained dried parts of the solutions and the respectively obtained crystals were measured through these techniques.

*Near Infrared Spectroscopy (NIR):* Thermo Scientific™ Antaris™ II FT-NIR Analyzer (Madison, WI, USA) was used qualitatively in order to detect any changes in the structure. The solid sample was loaded in the sample holder, and spectra were recorded from 4000 to 10,000 cm^−1^ with 8 cm^−1^ spectral resolution, and 32 scans were accumulated every time to improve the signal-to-noise ratio [16]. Background data were collected every half an hour. All spectra were acquired at room temperature. 

*Fourier-transform infrared spectroscopy (FTIR):* JASCO FTIR6300 spectrometer (Tokyo, Japan) was used for iron crystals with 0.07 cm^−1^ resolution in which the samples were pulverized, then mixed with KBr and pressed into tablets before measurement; the spectra were recorded from 400 till 4000 cm^−1^ [17,18]. 

### 2.5. Statistical Analyses

Statistical analyses for the quantitative results were done using IBM SPSS Statistics version 23.0. The primary outcome of the analyses was to check if the difference between the samples was statistically significant. One-way ANOVA was used to determine whether there were significant differences between the means of the four samples having different ratios. Post-hoc test (Tukey HSD^a^) was conducted after getting a significant difference to know where this difference truly came from. Values of *p* < 0.05 were taken as being statistically significant. Results are presented as Supplementary Materials.

## 3. Results and Discussion

### 3.1. Observations and Melting Point Results

Ferrous sulfate alone and all citric acid iron samples crystallized, whereas citric acid alone did not crystallize in the same conditions as the other samples. 

A melting point for all the crystals and the dried solutions was not achieved within the 300 °C range. All the samples did not melt in the 300 °C range, and only the color kept changing until becoming black. Whereas in the case of the dried solution of the citric–iron sample of ratio 2:1, it melted within 150–153 °C, which was the same range where the commercial citric acid alone melted, knowing that this range is recognized for pure citric acid [19]. This indicates the presence of free citric acid as opposed to the other samples in which citric acid is in the chelate form. Since citric acid alone did not crystallize, and due to its very high solubility in water, the excess citric acid remains soluble in the solution, as in the case of sample 2:1.

### 3.2. Quantitative Analyses (HPLC, AAS, and UV-Vis Spectroscopy)

**Figure 2:**
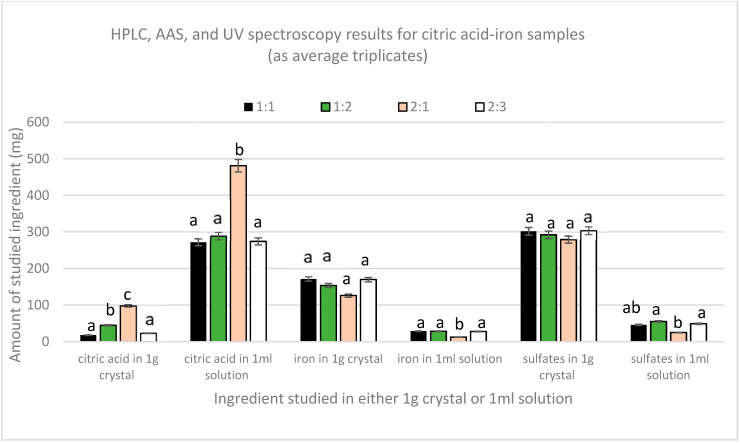
Average amount (mg) of citric acid, iron, and sulfates in crystal (1 g) or solution (1 mL).

Figure 2 shows the results obtained from HPLC, AAS, and UV-Vis spectroscopy for both the crystal and the remaining solution. Quantification of citric acid by HPLC has shown that the amount of citric acid in all the crystals was very low compared to the amount in the remaining solutions. The highest amount of citric acid in solutions was found to be in 2:1 sample, which was significantly different (p_(2:1,1:1)_ = 0.001 and p_(2:1,1:2)and (2:1,2:3)_ = 0.002) from the other three samples that had comparable amounts (p_(1:1,1:2)_ = 0.703, p_(1:2,2:3)_ = 0.742, and p_(1:1,2:3)_ = 0.958). In crystals, the 2:1 sample also contained the highest amount of citric acid, whereas the 1:1 and 2:3 had the lowest. Moreover, AAS results showed that a major part of iron was in the crystals and that the amount of iron in all the solutions was lower. The lowest amount of iron per ml found in sample 2:1 was significantly different from the other three samples, which contained similar amounts of iron per ml of solution (p_(2:1,1:1)_, p_(2:1,1:2),_ and p _(2:1,2:3)_ = 0.00). Nonetheless, UV spectroscopy revealed that sulfates were mainly present in the crystals. The solutions had a lower amount, with the 2:1 sample containing the lowest amount compared to the other three samples that have equal amounts per mL (p_(2:1,1:1)_ = 0.013, p_(2:1,1:2)_ = 0.002, and p _(2:1,2:3)_ = 0.007). 

From these results, we can consider that these crystals are impure crystals of FeSO_4_. This is further supported by the crystallization of pure ferrous sulfate without citric acid when following the same protocol, whereas citric acid alone did not crystallize. 

Nonetheless, due to the fact that the solutions contain both citric acid and iron, chelation could have been achieved in the liquid form. The 2:1 sample contains the highest amount of citric acid but the lowest amount of iron per ml solution if compared with the other three solutions. This indicates that in the case of the higher amount of citric acid, chelation is limited, and instead, citric–citric interactions are favored since excess ferrous sulfate has crystallized in this sample too. So chelation here is limited not due to lack of iron but due to the preference of citric acid to form dimers [20]. This verifies the melting point analyses in which all the samples did not melt in the 300 °C range, except the dried part of the 2:1 solution, which melted within the 150–153 range, indicating the presence of free citric acid. 

In the other three solutions, the molar proportionality between citric acid and iron in the solution is the same, regardless of the initial ratio (p _citric acid_ = 0.881 and p _iron_ = 0.878), in which approximately for every three moles of citric acid present there is one mole of iron. This proves that the relation between citric acid and iron is not affected by the initial amounts except in the case of the higher amount of citric acid, as in the 2:1 sample in which eleven moles of citric acid were found with one mole of iron. To further prove and validate this finding, new citric acid–iron samples were produced with significantly different molar ratios but in the less concentrated form to avoid the precipitation of iron that could have happened as a matter of solubility; the new trial is detailed below.

### 3.3. Spectral Analyses

#### 3.3.1. Fourier Transform Infrared Analyses (FTIR)

**Figure 3:**
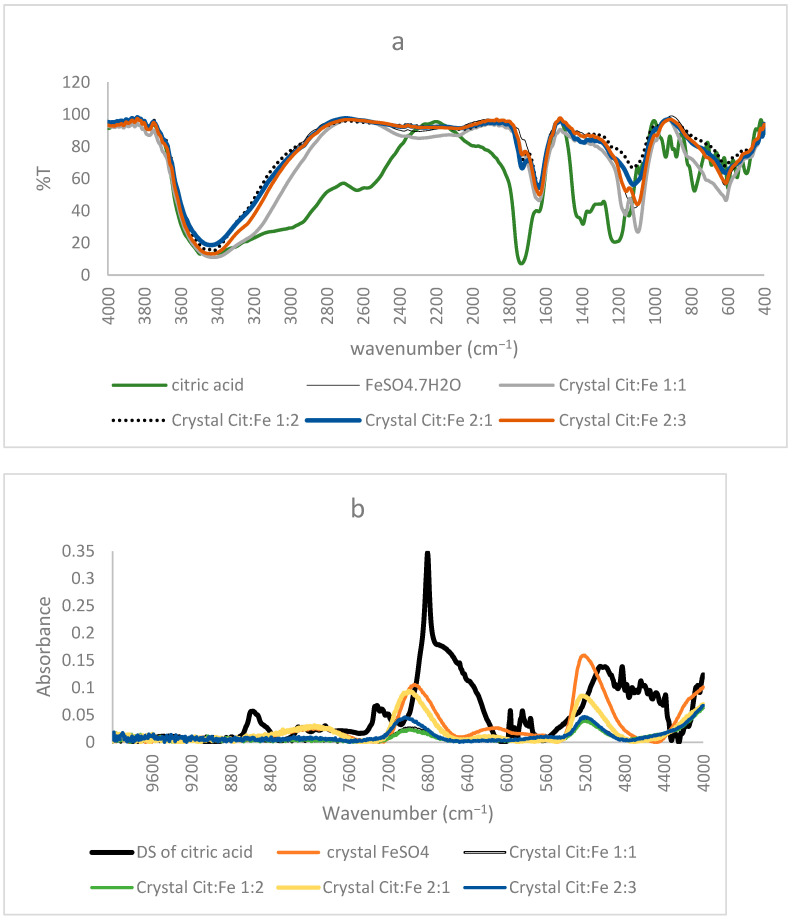
(**a**) FTIR, (**b**) NIR spectra of citric acid, FeSO_4_·7H_2_O, and the obtained crystals of citric–iron at ratios 1:1, 1:2, 2:1, and 2:3.

The importance of FTIR spectra in assigning peaks to functional groups is very well-known. Our finding that the obtained crystals are not chelates, as supposed by Yunarti et al. [5], but are, rather, the excess iron sulfates, is further supported by the results of FTIR, in which the spectra of all four crystals coincided with the pure form of FeSO_4_, in which all the peaks present in the crystal were also found in the commercial FeSO_4_. Thus, the absence of citric acid in the crystals proved by HPLC is further enhanced through the absence of any peaks corresponding to its functional group (C=O, O–H) or its structural chain (C–C, C–H) in the FTIR spectra of the crystals [9]. Moreover, the peak at 511 cm^−1^ assigned to Fe–O by [5] and used to prove the chelation of iron to the carboxyl group appeared in the spectra of the pure form of FeSO_4_. Nonetheless, [21] showed that the peaks in this region were linked to Fe–O vibrations in magnetite and hematite, further demonstrating the inaccuracy of relying on this peak to prove iron chelation (Figure 3a). 

#### 3.3.2. Near Infrared Analyses

**Figure 4:**
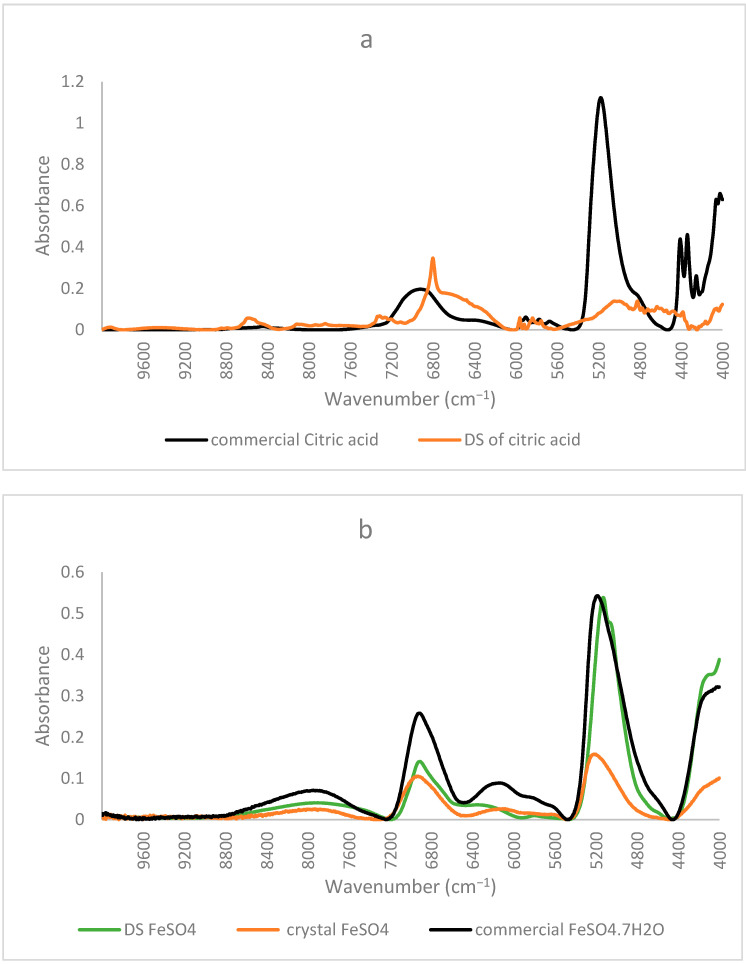
NIR spectra of commercial form and the dried solution of (**a**) citric acid; (**b**) iron sulfate with obtained crystal.

Similar to the FTIR results mentioned before, NIR spectra of ferrous sulfate and the citric–iron crystals also coincided, further proving them as excess ferrous sulfate (Figure 3b). NIR spectra of commercial citric acid and the dried solution of citric acid that underwent the same treatment as the other samples show important differences (Figure 4a). This could be due to the structural differences between the two and the formation of inter and intra-bonding (Figure 4b). Whereas the spectra of commercial iron sulfates and that of the dried solution are the same. This could be due to their inorganic nature and their ionic bonding not being detected in the NIR region in contrast to the organic nature of citric acid and the covalent bonding tending to form different conformations detected by near-infrared [16,22]. Thus, the spectra of the dried solution of citric acid will be used in the present analyses to eliminate any external variables and to be able to make a well-established comparison. 

**Figure 5:**
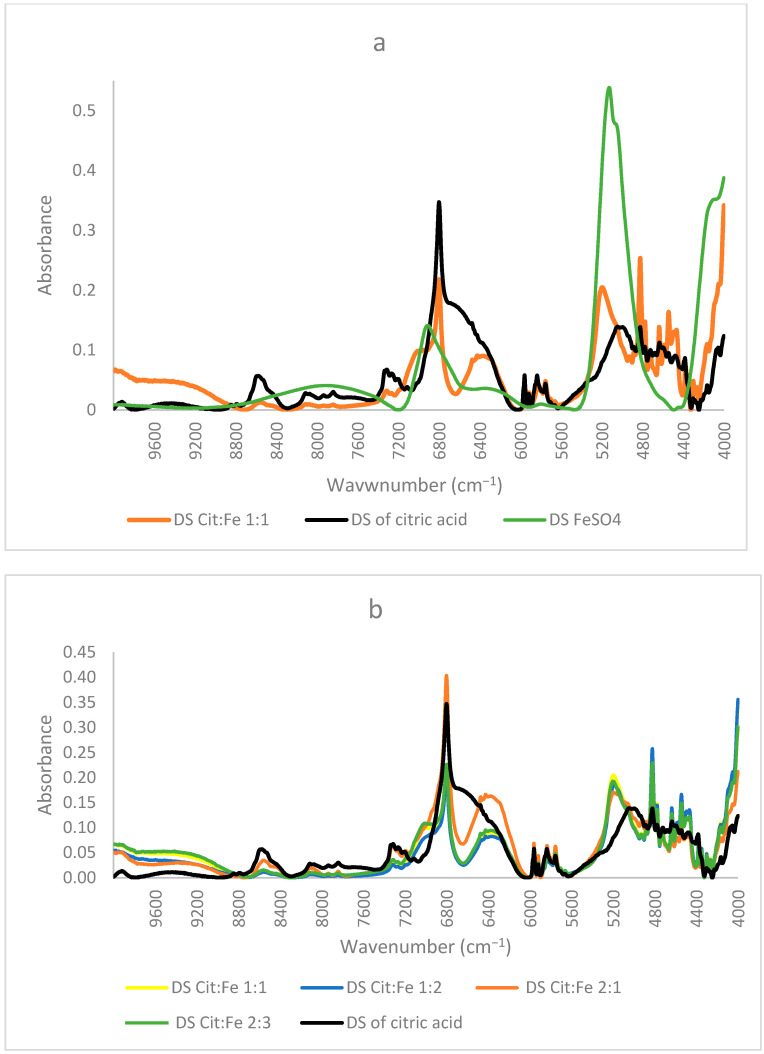
NIR spectra of dried solutions of (**a**) citric acid, iron sulfate, and citric:iron 1:1 sample; (**b**) the samples with different ratios.

Figure 5a shows the corresponding NIR spectra of dried solutions of citric acid alone, iron sulfate alone, and of the sample citric–iron in 1:1 ratio. It is obvious that the spectrum of the sample solution is different from those of both citric acid and iron sulfate alone. The spectrum of iron sulfate alone is totally different from the others presented with two peaks only at about 5138 cm^−1^ and 6920 cm^−1^. Whereas the sample and citric acid alone show some similarities in specific regions of the spectra, this is necessary and could be due to the skeletal structure that they both incorporate. Nonetheless, significant changes are observed in different regions.

In the region 10,000–6000 cm^−1^, most peaks are present in both spectra with changes in their intensities. The intensity of all the peaks significantly decreased in the CA:Fe 1:1 sample, except for a shoulder at about 7020 cm^−1^ and a broad peak centered at around 6370 cm^−1^, newly formed in the spectra of citric acid with iron. This shoulder could be due to the effect of the peak present in ferrous sulfate at 6920 cm^−1^. The region (10,000–6000 cm^−1^) is normally known for C–H combinations first overtone, C–H second overtone (10,000–7100 cm^−1^), and O–H first overtone (7100–6000 cm^−1^) [23]. Hence, the main differences could be in the structure of citric acid affecting C–H and C–H combinations. The peak at around 6800 cm^−1^ was very intense and sharp in citric acid; in the sample it remained sharp, but the intensity decreased. This peak corresponds to the O–H first overtone indicating the change that occurred in the functional group of citric acid due to the presence of iron. The broad peak centered at around 6370 cm^−1^ is present in neither the dried solution of citric acid alone nor in that of ferrous sulfate. Gandara et al. [24] mentioned that this peak is assigned to a charge transfer transition associated with the radical moiety and that in samples possessing this peak, the donor and acceptor orbitals are principally developed on the peripheral rings of the ligand. It was also mentioned that this peak corresponds to ligand-to-ligand charge transfer. Thus, this peak could show important information regarding the occurrence of citric acid–iron chelation.

The region 6000–4000 cm^−1^ in the spectra represents the region of noticeable changes; this region is the first overtone region for C–H and S–H (6000–5650 cm^−1^), the second overtone for C=O stretching (5556–5263 cm^−1^) and the combination regions of O–H (5263–4762 cm^−1^), C–H + C–H (4545–4200 cm^−1^), and C–H + C–C (4200–4000 cm^−1^). In the region of 6000–5500 cm^−1^, the four peaks were present in citric acid at about 5960, 5916, 5835, and 5754 cm^−1^, referring to CH_2_ (first overtone) [23]; they also appear in the sample of citric–iron and are the same. These peaks must refer to a part that remains intact; this means that they indicate the skeleton of citric acid not participating in the chelation. The clearest differences could be seen in the region between 5500–4000 cm^−1^. It is known that the peak centered at 5190 cm^−1^ corresponds to water, mainly bound water [25,26], but in our spectra, citric acid alone has a peak centered at 5020 cm^−1^ with the lowest intensity, iron sulfate alone has the highest intensity peak centered at 5138 cm^−1^, whereas the citric–iron sample has a peak with medium intensity at 5200 cm^−1^. If this peak is assigned to water, then it must be at the same wavenumber, as was mentioned by Padalkar [26], as no shift was observed for this peak. These changes in both the intensities and position are especially important in this region since within this wavenumber range there is a C=O stretching (second overtone), and the combination of the OH; thus, it is where the functional group of citric acid appears through NIR and where the reaction occurs. This fits well with the previous research and is in agreement with the published literature [22,27]. Moreover, [16] used NIR to differentiate between Fe(II) and Fe(III) and found that the peaks around 5150 cm^−1^ correspond to linkages with Fe(II), thus confirming that the iron chelated is still in its ferrous state. 

Moreover, many small, sharp peaks could be observed in the region of 4900–4300 cm^−1^. Peaks of citric–iron spectra are sharper and more pronounced. This region is known mainly for C–H + C–H, C–H +C–C and OH combinations [23,28,29]. Thus, these peaks show that structural organization has occurred inside the molecule, changing the conformation of the citric acid. Nonetheless, peaks at 4822, corresponding to in-phase bending of OH, at 4777 to OH combination, 4635 and 4584 to C–C combination, 4542 to CHO combination, and 4347, 4300, 4262, and 4163 cm^−1^ to CH, are all sharper and more pronounced in the spectra of the citric–iron sample as compared with citric acid alone, thus further indicating changes in citric acid in the functional group and the neighboring atoms. The peaks at 4723, 4495, and 4464 cm^−1^ are similar in both spectra and correspond to the CH_2_ combination, further indicating that the skeleton of citric acid stayed unaltered [23,28,30]. 

Figure 5b shows the NIR spectra of the dried solution of citric acid and the dried solutions of citric acid–iron samples in different ratios. It is clear that the spectra of the ratios 1:1, 1:2, and 2:3 are very similar and are coinciding in mostly all regions, with the only observed changes in the slight increase or decrease in the intensities. This fits well with the previously mentioned quantitative results stating that the three solutions contain equal amounts of citric acid, iron, and sulfates. In contrast, when comparing these three with the spectra of the 2:1 sample, some similarities appear, but differences are also observed. This latter sample shows more similarities with the spectra of the dried solution of citric acid than the other three. It appears as a mixture of a region similar to the dried solutions of the other ratios and another region similar to that of citric acid alone. The region between 10,000 and 6750 cm^−1^ in the spectrum of the 2:1 sample resembles citric acid, whereas in the other part of the spectrum, it is similar to the other ratios but has the highest intensity for the peak at 6370 cm^−1^ assigned before for the ligand-to-ligand charge transfer [24]. This indicates that the formation of dimers in this sample is higher compared to the other samples, which could validate our suggestions in the explanation that this sample has the lowest amount of iron remaining in the solution based on the quantitative results due to the preference of citric acid dimer formation. Thus, from all the above findings and based on the comparison of peaks in the present work with those in the investigations of other authors, we can confirm that three peaks at 6800, 6370, and 5150 cm^−1^ can be used to know the direction of the reaction and to prove iron chelation. Moreover, NIR, which is already extensively used in the food and pharmaceutical industries, proved to be very useful in proving chelation. 

**Figure 6:**
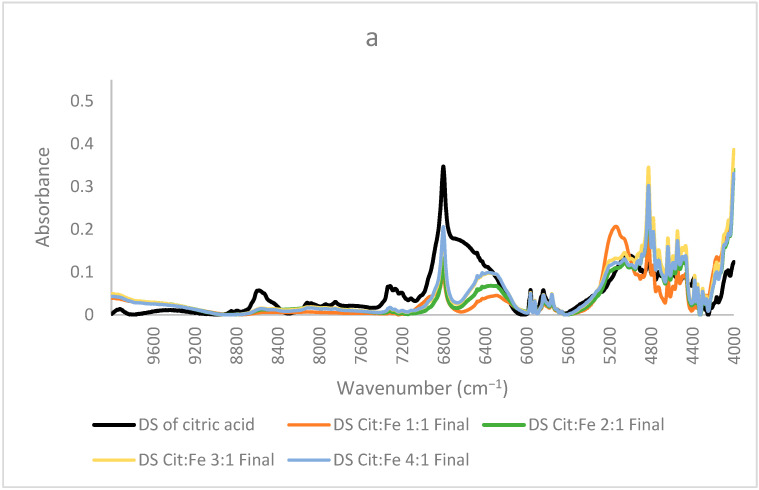

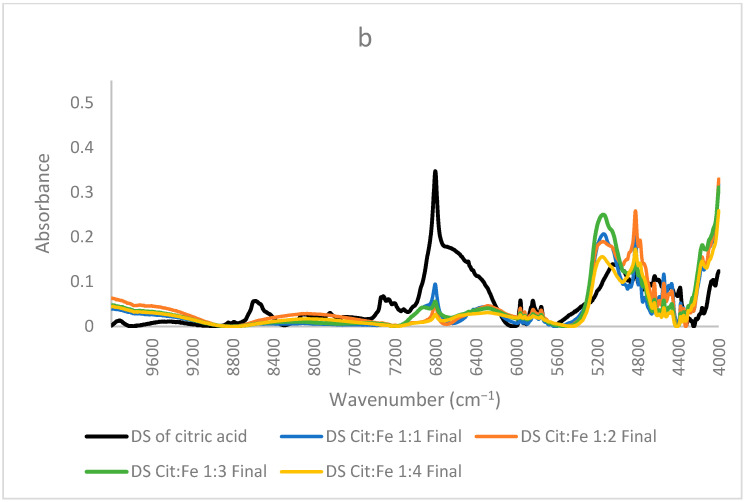
NIR spectra of dried solutions of citric acid and samples prepared at lower concentrations (**a**) ratios 1:1,2:1, 3:1,4:1 (**b**) 1:1, 1:2, 1:3 and 1:4.

**Study of Molar Ratio:** In order to understand the effect of molar ratio on chelation and whether to prove or not the fixed ratio of 3:1 obtained from the quantitative analyses, new citric acid–iron samples were produced. These samples have significantly different molar ratios but in a less concentrated form. In these samples, crystallization did not occur, which further proves that the precipitation of iron has happened as a matter of its low solubility. Moreover, this trial might help us indicate the presence of free citric acid remaining in the solution due to its high solubility and to find the optimum ratio for citric acid–iron chelation. Figure 6a shows the NIR spectra of the dried solution of the samples (1:1, 2:1, 3:1, and 4:1) with the spectrum of the dried citric acid solution. The overall spectra share similar peaks with the dried solutions of the concentrated samples discussed before. In these spectra, three important peaks of 6800, 6370, and 5150 cm^−1^ are our main concerns. The intensity of the peak corresponding with O–H first overtone at 6800 cm^−1^ increased with citric acid, in which the 4:1 sample had the highest intensity, whereas the 1:1 sample had the lowest intensity. This indicates the presence of free citric acid and free O–H sites. Similarly, the peak at 6370 cm^−1^ increased in the same order when adding more citric acid. This peak was assigned to the ligand-to-ligand radical charge transfer transition by [24], and it is the most intense in the sample containing the highest amount of citric acid. This validates that citric–citric interactions occur through the transfer of charge. The higher the amount of citric acid, the more interactions occur. The third peak at about 5150 cm^−1^ is in the region corresponding to stretching of C=O and the combination of O–H mainly for water. A significant decrease for this peak is observed between the spectrum of the 1:1 sample and the other three samples (2:1, 3:1, and 4:1). The three samples were dried the same way, so this difference must be interpreted for something other than a water amount. Since this region is also observed for C=O, upon having higher amounts of citric acid, the carbonyl group might have been appearing higher. Nonetheless, this peak could be assigned to the free O–H, which is high in the 1:1 sample, whereas in the other samples and due to the possibility of dimer formation between citric acid molecules, its intensity decreased. This fits well with the interpretation of the peak at 6370 cm^−1^ of the ligand-to-ligand charge transfer described by [24], where the least intense peak is for 1:1 sample, revealing that dimer formation is enhanced in the other three samples having the higher amounts of citric acid. 

In contrast, Figure 6b shows the spectra of the samples, where the citric acid amount is fixed, and the amount of iron sulfate is increased (1:1, 1:2, 1:3, and 1:4). The same three peaks will be focused on since the other peaks are more related to the structure and are similar in most samples. In these samples, the only difference is the amount of iron sulfate the peak at 6800 cm^−1^ decreased remarkably, showing the decrease in the free O–H bonds and, giving the proof of iron chelation, this decrease could be well described by the findings of [31], who stated that citric acid chelates iron not only by its carboxylic groups but also by involving its hydroxyl group. Thus, the decrease in the free OH is clearly interpreted. Sample 1:4 has the lowest intensity both in this peak and the peak corresponding to ligand–ligand interactions at 6370 cm^−1^ (almost disappearing), further validating the occurrence of chelation and the interaction of iron with citric acid as opposed to citric–citric interactions occurring in all the other samples in a different scale according to each. Similarly, this sample has the lowest intensity also for the peak at 5150 cm^−1^ corresponding to free O–H bonds and stretching of C=O; the other samples have higher intensities, with sample 1:3 acquiring the highest intensity. Sample 1:2 has a lower intensity than 1:1, but 1:3 has a higher intensity than both. This difference in the order could be due to the effect of the ratio forcing some configurations to be different from the other two samples. Because this peak is for the in-phase bending of O–H and C=O stretching [32], this ratio is the only odd ratio, which might have been causing O–H and C=O to, respectively, bend and stretch more as compared to the other ratios. Moreover, (31) has validated that the mode of citric acid chelation varies with the form of iron in which it behaves as a tridentate with ferrous involving its hydroxyl group and a bidentate with ferric through their carboxylic groups only. This inference goes in the same direction as the interpretation of the peaks assigned to the functional groups of citric acid. Therefore, from all these findings, we can state that the ratio 1:4 has no free citric acid remaining in the solution, and no dimer formation has taken place, whereas the 1:3 ratio behaves differently and needs further investigation. It is worth mentioning that the chelation of iron by citric acid has taken place even at low pH (around 1). This goes well with the study conducted by [33], in which they found that citric acid at pH 1 is the best for chelation compared to EDTA, EGTA, CDTA, and citric acid at higher pH values. These results are highly valuable if adopted to produce a new stable and affordable food fortifier to be either added to food in order to increase its iron content or to be formulated into iron supplements. 

## 4. Conclusions

A new stable food fortifier based on the chelation of iron by citric acid was produced. The molar ratio appeared to be fundamental in determining the reaction. NIR, which is present in almost every industry, has proved to be a very useful technique to confirm the occurrence of chelation. Three peaks from NIR spectra at 6800, 6370, and 5150 cm^−1^ (corresponding to O–H first overtone, ligand-to-ligand radical charge transfer transition, and stretching of C=O and combination of O–H, respectively) could be used to characterize the obtained chelates. It is important to produce the chelates within the solubility range to avoid any unnecessary crystallization of the excess. The molar ratio Citric:Fe 1:4 proved to be the most optimum one. These findings are especially important for food and pharmaceutical industries in which they could be applied to produce an iron fortificant to be either added to food or formulated into supplements. This fortifier is stable, easy to produce within the industry, and affordable, thus increasing the iron content without a huge increase in the price of the respective fortified components. Future studies should focus on studying the structure of these chelates, and knowing the exact conformation between citric acid and iron, XRD, and EXAFS, among others, is needed so that we can confirm whether iron is being chelated by the same citric acid molecule or by more than one. Investigations about the bioavailability of these citric acid–iron chelates, as well as the behavior of other minerals, such as zinc and magnesium, with citric acid or different ligands, are now in progress and will be published in the near future.

## Figures and Tables

**Table 1:** Percentage recovery values of citric acid standard.

Standard	% Recovery
0.1	124.08
0.5	100.1
2	98.16
5	99.05
10	100.3

## Data Availability

Data is contained within the article or supplementary material.

## Supplementary Materials

The following supporting information can be downloaded at: https://www.mdpi.com/article/10.3390/foods12020410/s1.
