# Supplementary material for: Are Citric Acid-Iron II Complexes True Chelates or Just Physical Mixtures and How to Prove This?
Source: Foods. 2023 Jan 15;12(2):410. doi: 10.3390/foods12020410 (PMC9858486; doi:10.3390/foods12020410)
Supplement: Supplementary file 1 [file foods-12-00410-s001.zip › foods-2144901-supplementary.pdf]

```
ONEWAY citricacidinsolution citricacidincrystal ironinsolution ironincrystal s
ulfateinsolution
    sulfateincrystal BY samplerep
  /MISSING ANALYSIS
  /POSTHOC=TUKEY ALPHA(0.05).
```

Oneway

ANOVA

		Sum of Squares	df	Mean Square	F	Sig.
citricacidinsolution	Between Groups	9.310	3	3.103	10.677	.004
	Within Groups	2.325	8	.291		
	Total	11.636	11			
citricacidincrystal	Between Groups	120.392	3	40.131	73.078	.000
	Within Groups	4.393	8	.549		
	Total	124.785	11			
ironinsolution	Between Groups	551.674	3	183.891	32.982	.000
	Within Groups	44.604	8	5.576		
	Total	596.278	11			
ironincrystal	Between Groups	4037.391	3	1345.797	2.110	.177
	Within Groups	5103.167	8	637.896		
	Total	9140.557	11			
sulfateinsolution	Between Groups	1572.860	3	524.287	7.828	.009
	Within Groups	535.837	8	66.980		
	Total	2108.697	11			
sulfateincrystal	Between Groups	1152.751	3	384.250	.893	.486
	Within Groups	3444.134	8	430.517		
	Total	4596.885	11			

Post Hoc Tests

Multiple Comparisons

Tukey HSD

Dependent Variable	(I) samplerep	(J) samplerep	Mean Difference (I-J)	Std. Error	Sig.	95% Confidence Interval	
						Lower Bound	Upper Bound
citricacidinsolution	11	12	-.17420	.44019	.978	-1.5838	1.2355
		21	-2.09436*	.44019	.006	-3.5040	-.6847
		23	-.02389	.44019	1.000	-1.4335	1.3858
	12	11	.17420	.44019	.978	-1.2355	1.5838
		21	-1.92017*	.44019	.010	-3.3298	-.5105
		23	.15030	.44019	.985	-1.2593	1.5600
	21	11	2.09436*	.44019	.006	.6847	3.5040
		12	1.92017*	.44019	.010	.5105	3.3298
		23	2.07047*	.44019	.007	.6608	3.4801
	23	11	.02389	.44019	1.000	-1.3858	1.4335
		12	-.15030	.44019	.985	-1.5600	1.2593
		21	-2.07047*	.44019	.007	-3.4801	-.6608
citricacidincrystal	11	12	-2.71919*	.60506	.009	-4.6568	-.7816
		21	-8.02930*	.60506	.000	-9.9669	-6.0917
		23	-.57685	.60506	.778	-2.5145	1.3608
	12	11	2.71919*	.60506	.009	.7816	4.6568
		21	-5.31011*	.60506	.000	-7.2477	-3.3725
		23	2.14235*	.60506	.031	.2047	4.0800
	21	11	8.02930*	.60506	.000	6.0917	9.9669
		12	5.31011*	.60506	.000	3.3725	7.2477
		23	7.45245*	.60506	.000	5.5148	9.3901
	23	11	.57685	.60506	.778	-1.3608	2.5145
		12	-2.14235*	.60506	.031	-4.0800	-.2047
		21	-7.45245*	.60506	.000	-9.3901	-5.5148
ironinsolution	11	12	.20000	1.92796	1.000	-5.9740	6.3740
		21	15.99167*	1.92796	.000	9.8177	22.1657
		23	.85000	1.92796	.970	-5.3240	7.0240
	12	11	-.20000	1.92796	1.000	-6.3740	5.9740
		21	15.79167*	1.92796	.000	9.6177	21.9657
		23	.65000	1.92796	.986	-5.5240	6.8240
	21	11	-15.99167*	1.92796	.000	-22.1657	-9.8177
		12	-15.79167*	1.92796	.000	-21.9657	-9.6177
		23	-15.14167*	1.92796	.000	-21.3157	-8.9677
	23	11	-.85000	1.92796	.970	-7.0240	5.3240
		12	-.65000	1.92796	.986	-6.8240	5.5240
		21	15.14167*	1.92796	.000	8.9677	21.3157
ironincrystal	11	12	18.16667	20.62193	.815	-47.8720	84.2053
		21	45.91667	20.62193	.196	-20.1220	111.9553
		23	2.16667	20.62193	1.000	-63.8720	68.2053
	12	11	-18.16667	20.62193	.815	-84.2053	47.8720
		21	27.75000	20.62193	.563	-38.2887	93.7887
		23	-16.00000	20.62193	.863	-82.0387	50.0387
	21	11	-45.91667	20.62193	.196	-111.9553	20.1220
		12	-27.75000	20.62193	.563	-93.7887	38.2887
		23	-43.75000	20.62193	.225	-109.7887	22.2887

Multiple Comparisons

Tukey HSD

Dependent Variable	(I) samplerep	(J) samplerep	Mean Difference (I-J)	Std. Error	Sig.	95% Confidence Interval	
						Lower Bound	Upper Bound
	23	11	-2.16667	20.62193	1.000	-68.2053	63.8720
		12	16.00000	20.62193	.863	-50.0387	82.0387
		21	43.75000	20.62193	.225	-22.2887	109.7887
sulfateinsolution	11	12	-9.35758	6.68229	.533	-30.7566	12.0415
		21	21.13128	6.68229	.053	-.2678	42.5303
		23	-3.04184	6.68229	.967	-24.4409	18.3572
	12	11	9.35758	6.68229	.533	-12.0415	30.7566
		21	30.48886*	6.68229	.008	9.0898	51.8879
		23	6.31574	6.68229	.783	-15.0833	27.7148
	21	11	-21.13128	6.68229	.053	-42.5303	.2678
		12	-30.48886*	6.68229	.008	-51.8879	-9.0898
		23	-24.17312*	6.68229	.028	-45.5722	-2.7741
	23	11	3.04184	6.68229	.967	-18.3572	24.4409
		12	-6.31574	6.68229	.783	-27.7148	15.0833
		21	24.17312*	6.68229	.028	2.7741	45.5722
sulfateincrystal	11	12	9.56798	16.94140	.940	-44.6844	63.8203
		21	22.57005	16.94140	.570	-31.6823	76.8224
		23	-2.32988	16.94140	.999	-56.5822	51.9225
	12	11	-9.56798	16.94140	.940	-63.8203	44.6844
		21	13.00207	16.94140	.867	-41.2503	67.2544
		23	-11.89786	16.94140	.893	-66.1502	42.3545
	21	11	-22.57005	16.94140	.570	-76.8224	31.6823
		12	-13.00207	16.94140	.867	-67.2544	41.2503
		23	-24.89993	16.94140	.496	-79.1523	29.3524
	23	11	2.32988	16.94140	.999	-51.9225	56.5822
		12	11.89786	16.94140	.893	-42.3545	66.1502
		21	24.89993	16.94140	.496	-29.3524	79.1523

*. The mean difference is significant at the 0.05 level.

Homogeneous Subsets

citricacidinsolution

Tukey HSD[a]

samplerep	N	Subset for alpha = 0.05	
		1	2
11	3	2.7176	
23	3	2.7415	
12	3	2.8918	
21	3		4.8120
Sig.		.978	1.000

Means for groups in homogeneous subsets are displayed.

a. Uses Harmonic Mean Sample Size = 3.000.

citricacidincrystal

Tukey HSD[a]

samplerep	N	Subset for alpha = 0.05		
		1	2	3
11	3	1.7742		
23	3	2.3510		
12	3		4.4934	
21	3			9.8035
Sig.		.778	1.000	1.000

Means for groups in homogeneous subsets are displayed.

a. Uses Harmonic Mean Sample Size = 3.000.

ironinsolution

Tukey HSD[a]

samplerep	N	Subset for alpha = 0.05	
		1	2
21	3	12.7333	
23	3		27.8750
12	3		28.5250
11	3		28.7250
Sig.		1.000	.970

Means for groups in homogeneous subsets are displayed.

a. Uses Harmonic Mean Sample Size = 3.000.

ironincrystal

Tukey HSD[a]

samplerep	N	Subset for alpha = 0.05
		1
21	3	126.0000
12	3	153.7500
23	3	169.7500
11	3	171.9167
Sig.		.196

Means for groups in homogeneous subsets are displayed.

a. Uses Harmonic Mean Sample Size = 3.000.

sulfateinsolution

Tukey HSD[a]

samplerep	N	Subset for alpha = 0.05	
		1	2
21	3	24.7923	
11	3	45.9236	45.9236
23	3		48.9654
12	3		55.2812
Sig.		.053	.533

Means for groups in homogeneous subsets are displayed.

a. Uses Harmonic Mean Sample Size = 3.000.

sulfateincrystal

Tukey HSD[a]

samplerep	N	Subset for alpha = 0.05
		1
21	3	279.0697
12	3	292.0718
11	3	301.6398
23	3	303.9696
Sig.		.496

Means for groups in homogeneous subsets are displayed.

a. Uses Harmonic Mean Sample Size = 3.000.
